# Pure Red Cell Aplasia Associated With Thymoma: A Case Report

**DOI:** 10.7759/cureus.115780

**Published:** 2026-09-04

**Authors:** Amine Benslimane, Layla Mohib, Soumaya El Yakoubi, Youssef Mahdi, Chaimaa Aboumedian, Roda Hassan Eltigani, Ibrahim El Ghissassi, Basma El Khannoussi, Hassan Errihani

**Affiliations:** 1 Department of Medical Oncology, National Institute of Oncology, Rabat, MAR; 2 Faculty of Medicine and Pharmacy, Mohammed V University, Rabat, MAR; 3 Department of Pathology, National Institute of Oncology, Rabat, MAR

**Keywords:** autoimmune disease, chemotherapy, immunosuppressive therapy, pure red cell aplasia, thymoma

## Abstract

A 55-year-old male, former smoker, presented with exertional dyspnea associated with an anterior mediastinal mass. Biopsy findings were consistent with a type B2 thymoma. The tumor was deemed unresectable, and curative concurrent chemoradiotherapy was not feasible after three cycles of cyclophosphamide, adriamycin (doxorubicin), and platinum (cisplatin) (CAP) chemotherapy. Treatment was therefore continued to six cycles. Following radiological progression, second-line chemotherapy with paclitaxel and carboplatin was initiated. After two cycles, the patient developed superior vena cava syndrome. Palliative mediastinal radiotherapy was subsequently delivered.

The patient experienced five episodes of severe hyporegenerative anemia, in the absence of bleeding or hemolysis, requiring repeated blood transfusions. Both the bone marrow aspirate and the bone marrow biopsy revealed pure red cell aplasia (PRCA). Testing for parvovirus B19 infection was negative.

Immunosuppressive therapy with prednisone, combined with third-line etoposide-based chemotherapy, was initiated. However, the patient discontinued treatment after two cycles and remained transfusion-dependent during follow-up. This case highlights the diagnostic and therapeutic challenges posed by thymoma-associated PRCA in patients with advanced unresectable disease.

## Introduction

Thymoma is a rare epithelial tumor of the thymus, accounting for 0.2% to 1.5% of malignant neoplasms, with an incidence of 0.13 to 0.32 per 100,000 per year. It is the most common tumor of the anterior mediastinum [1]. Although thymic tumors can occur at any age, they are most commonly diagnosed in individuals between 50 and 60 years of age [2]. Presenting symptoms are related to mediastinal compression in approximately 30% of cases and include cough, dyspnea, and chest pain [1,3]. Myasthenia gravis is found in 40% of cases, while other autoimmune disorders are reported in approximately 5% of cases [1]. These autoimmune manifestations are also known as parathymic syndromes. The curative treatment of thymoma is based on surgery, either alone or combined with radiotherapy and/or chemotherapy [3].

Anemia is a common symptom in cancer patients; it has various causes and is particularly common in patients undergoing chemotherapy. It is associated with asthenia and reduced quality of life and may have a negative impact on the response to oncologic treatment [4]. In the case of thymoma, anemia may occur as part of parathymic syndromes associated with red cell aplasia or hypoplasia, hemolytic anemia, or Biermer’s anemia [1]. Among these conditions, pure red cell aplasia (PRCA) is a rare thymoma-associated autoimmune syndrome characterized by an isolated absence of erythroid precursors in the bone marrow. It occurs in approximately 2% to 5% of patients with thymoma [2]. Thymectomy alone is not associated with a high rate of sustained remission of PRCA [5]. Immunosuppressive therapy is often required in addition to thymectomy to improve remission rates in patients with PRCA [6]. We report the case of a patient with advanced thymoma who developed PRCA, highlighting the diagnostic challenge of distinguishing this rare immune-mediated syndrome from other causes of anemia in patients with cancer, as well as the therapeutic challenges associated with its management.

## Case presentation

The patient is a 55-year-old male, a former chronic smoker, who presented with exertional dyspnea with preserved general condition. Chest CT (Figures 1-2) revealed a large anterior mediastinal mass with the longest diameter of 13 cm extending into the superior and middle mediastinum, with involvement of the supra-aortic vessels and aortic arch and compression of the superior vena cava. Associated findings included subcarinal and right interbronchial lymphadenopathy, moderate pericardial effusion, and right pleural thickening. A CT-guided biopsy of the mediastinal mass was performed. Histopathological examination of the biopsy specimen revealed a classic organoid appearance characteristic of a type B2 thymoma. The tumor showed a prominent mixture of abundant small, non-neoplastic lymphocytes (thymocytes) interspersed with clusters of large, polygonal neoplastic epithelial cells (Figure 3). Immunohistochemical profiling confirmed the diagnosis. The neoplastic epithelial cells exhibited strong, diffuse cytoplasmic positivity for AE1/AE3 (pan-cytokeratin). The background thymocytes were strongly positive for immature T-cell markers, including terminal deoxynucleotidyl transferase (TdT), CD5, and CD3 (Figure 4).

**Figure 1:**
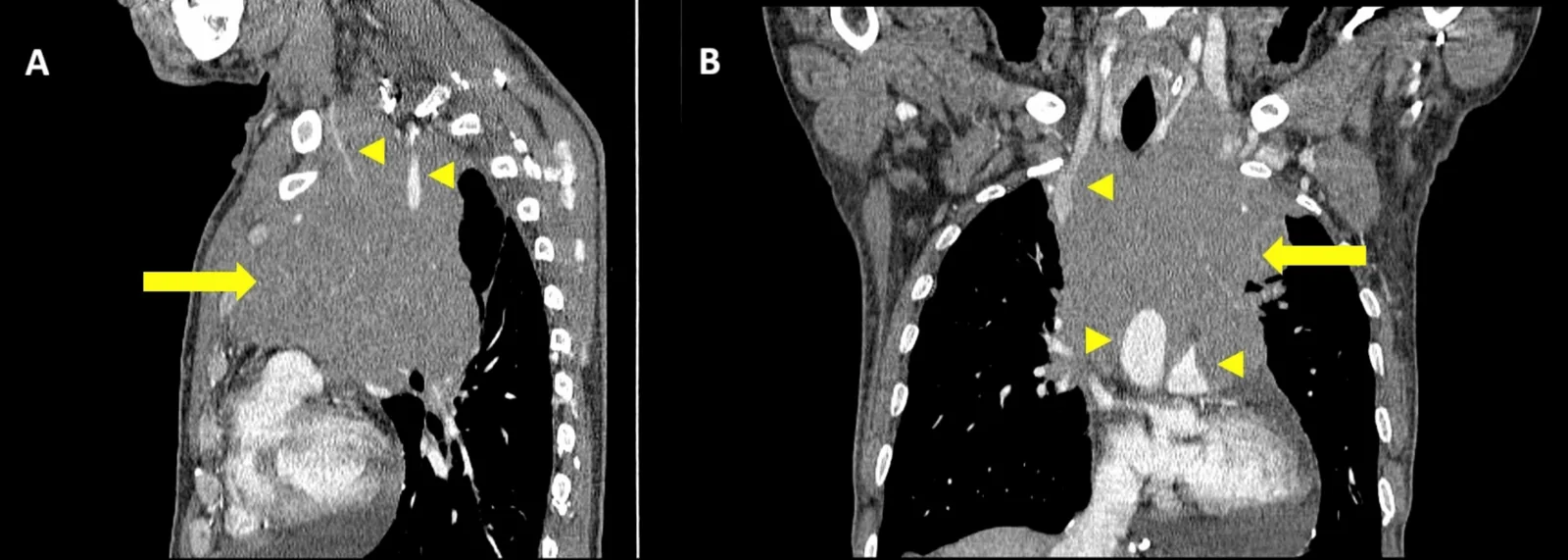
CT scan of the thymoma Sagittal (A) and coronal (B) views of the CT images demonstrate a locally advanced thymoma (yellow arrows) occupying the anterior mediastinum with involvement of the mediastinal vessels (yellow arrowheads).

**Figure 2:**
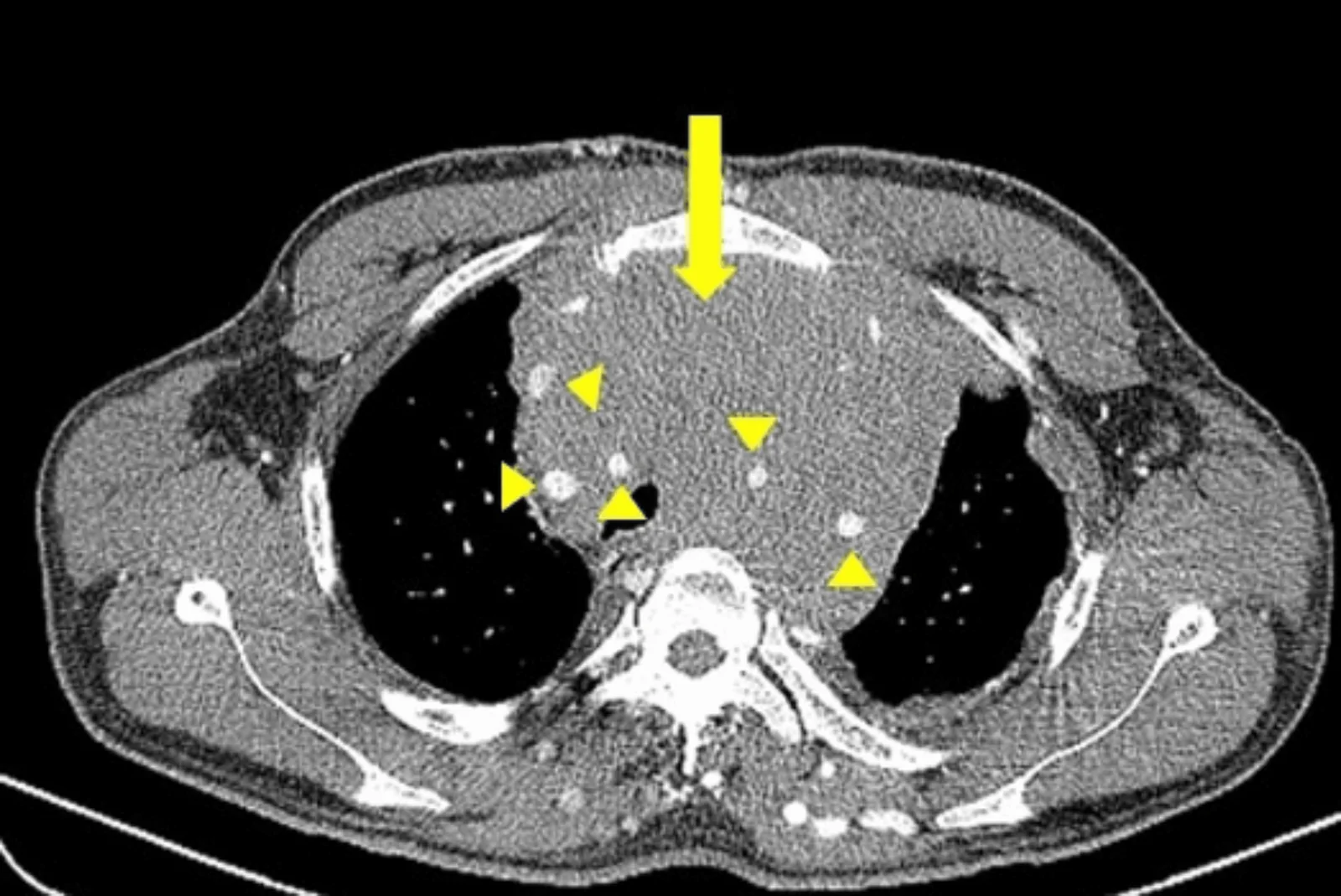
Axial CT image of the thymoma This axial CT image shows the thymoma (yellow arrow) with involvement of the supra-aortic vessels (yellow arrowheads).

**Figure 3:**
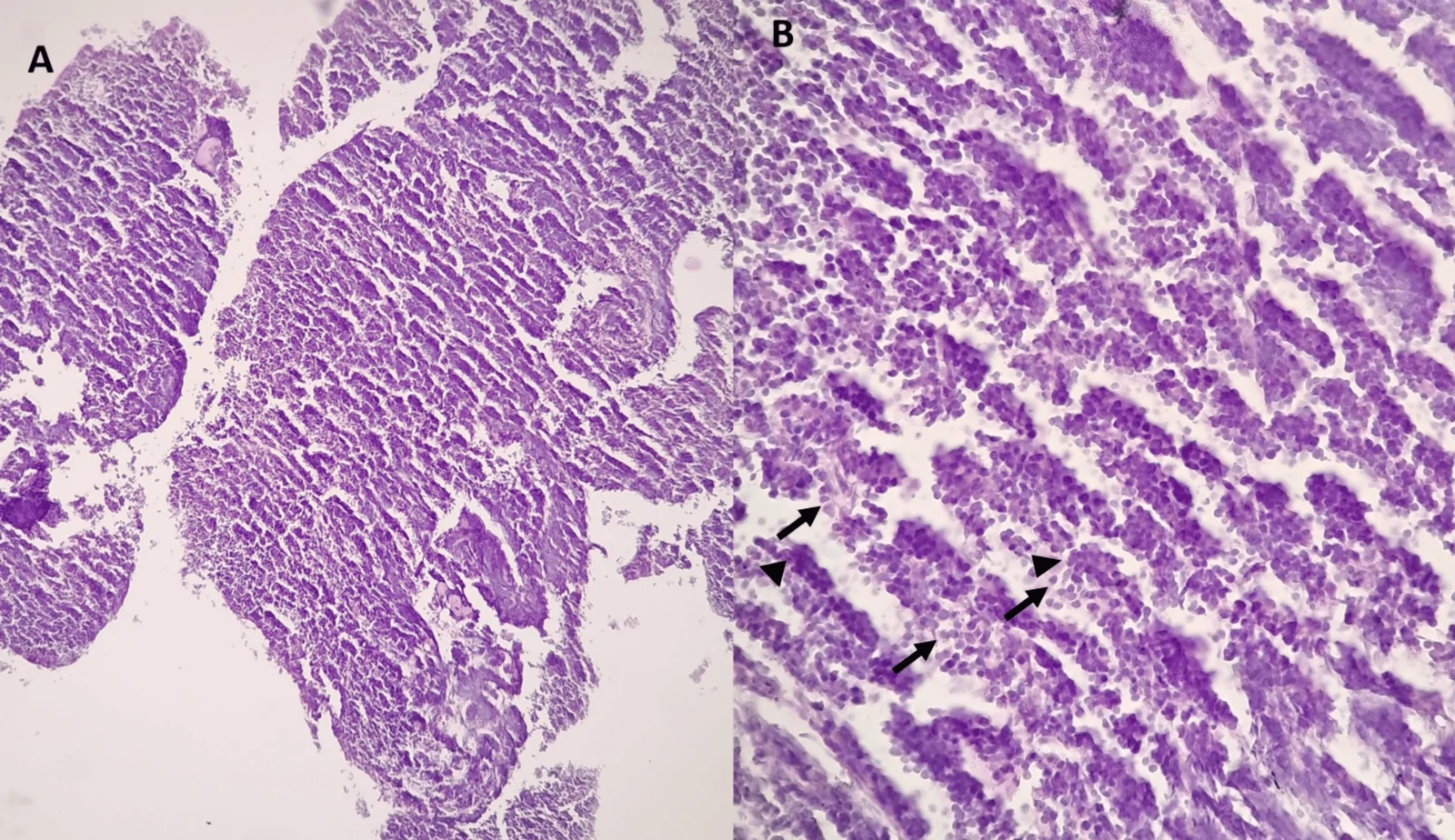
Representative micrograph of the tumor (thymoma B2) The tumor shows a mixture of small lymphocytes (thymocytes) marked by the black arrowheads and clusters of large polygonal neoplastic epithelial cells (black arrows). Hematoxylin-eosin: Panel A x100, panel B x400

**Figure 4:**
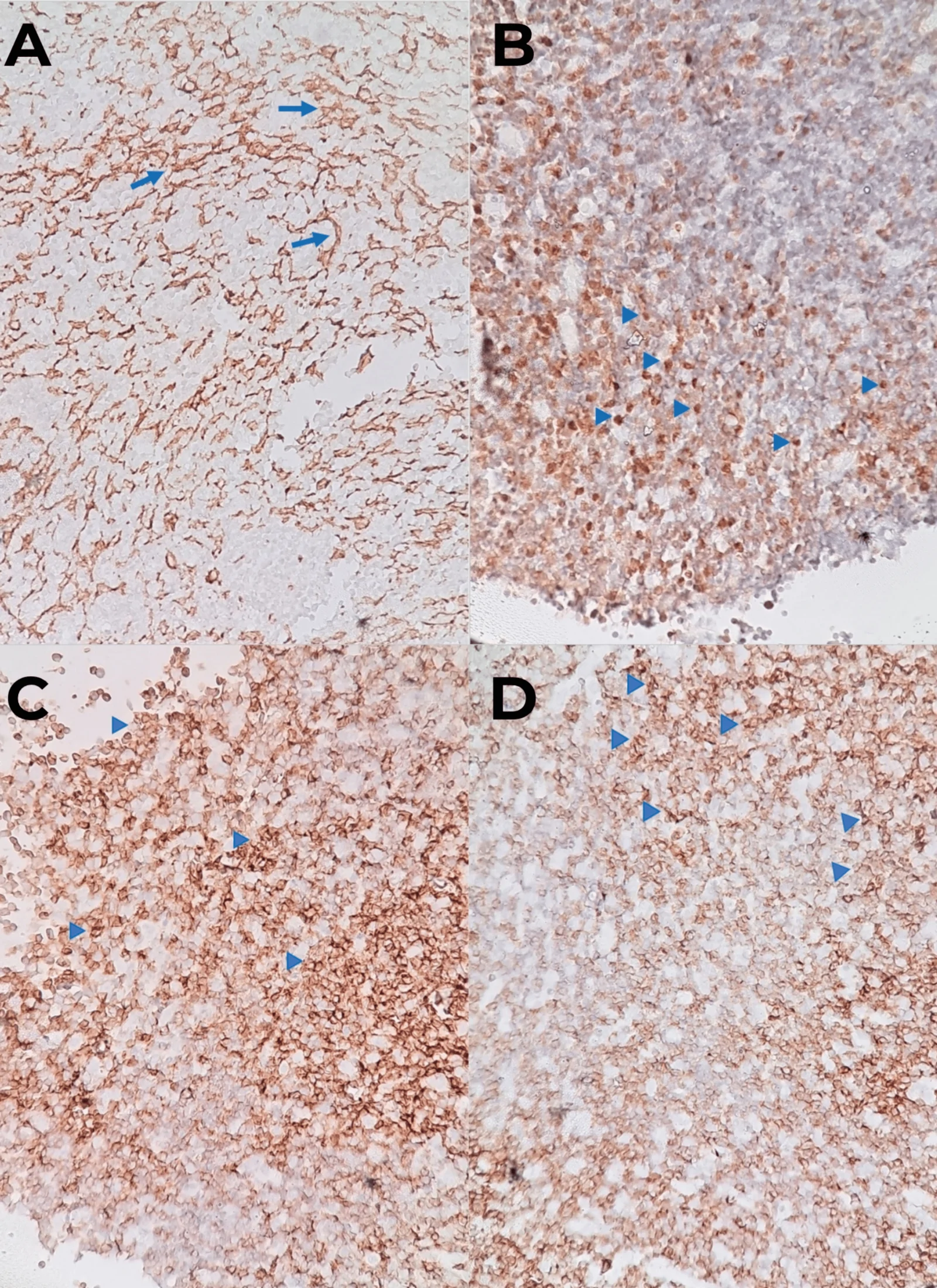
Immunohistochemistry profile of the tumor (thymoma B2) A: Neoplastic cells are positive for AE1/AE3 (pan-cytokeratin); B: Thymocytes are positive for TdT; C: CD5; D: CD3; Blue arrows: Epithelial cells; Blue arrowheads: Thymocytes (lymphocytes) (×400) TdT: Terminal deoxynucleotidyl transferase

Given the unresectable nature of the tumor, chemotherapy with the cyclophosphamide, adriamycin (doxorubicin), and platinum (cisplatin) (CAP) regimen was initiated, consisting of cisplatin (50 mg/m²), doxorubicin (50 mg/m²), and cyclophosphamide (500 mg/m²) every three weeks. Response evaluation after three cycles demonstrated stable disease. Since concomitant curative-intent chemoradiotherapy was not feasible, chemotherapy with the CAP regimen was continued until completion of six cycles. Evaluation after six cycles demonstrated progression of the mediastinal disease. Second-line chemotherapy with paclitaxel (175 mg/m²) and carboplatin at an area under the concentration-time curve (AUC) of 5 mg/mL × min administered every 21 days was initiated. After two cycles, the patient developed superior vena cava syndrome secondary to thymoma progression, demonstrated by CT imaging performed 72 days after the last cycle of chemotherapy. Palliative mediastinal radiotherapy (30 Gy in 10 fractions of 3 Gy each) was subsequently delivered, along with supportive measures including corticosteroids, anticoagulation, and oxygen therapy.

The clinical course was marked by five episodes of severe anemia, with hemoglobin (Hb) levels ranging from 3 g/dL to 5 g/dL, each requiring repeated blood transfusions. We considered day zero (D0) to be the date of the second cycle of paclitaxel-carboplatin. The first episode occurred on day 88, the second on day 134, the third on day 176 during mediastinal radiotherapy, which was delivered between days 168 and 179, the fourth on day 225, and the fifth on day 267. Figure 5 illustrates the timeline of the different episodes of anemia, the corresponding Hb levels, and the number of units of packed red blood cells (PRBCs) transfused during each episode.

**Figure 5:**
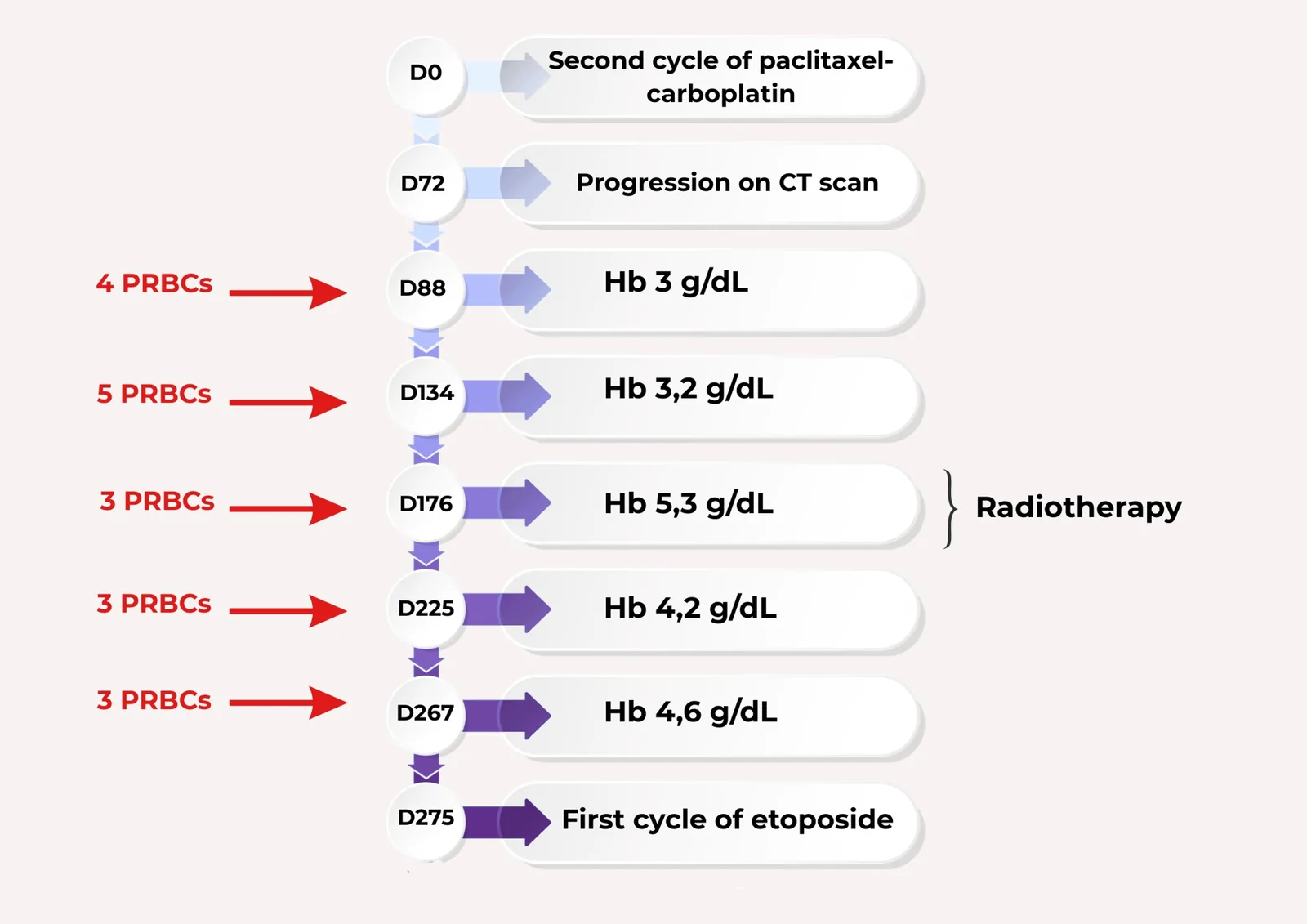
Timeline illustrating the occurrence of anemia episodes throughout the course of the disease D: Day; PRBCs: Packed red blood cells; Hb: Hemoglobin

Laboratory analyses revealed anemia with an Hb level of 3 g/dL (reference range: 13-16.5 g/dL), a mean corpuscular volume (MCV) of 86.7 µm³ (reference range: 80-100 µm³), a mean corpuscular hemoglobin (MCH) of 28.8 pg (reference range: 27-32 pg), and an absolute reticulocyte count of 7.8 × 10³/µL (reference range: 20-80 × 10³/µL). The findings were consistent with a non-regenerative normocytic, normochromic anemia. The remaining complete blood count parameters included a white blood cell count of 8.25 × 10³/µL (reference range: 4.0-10.0 ×10³/µl), with an absolute neutrophil count of 4.24 × 10³/µL (reference range: 1.5-7 ×10³/µl), and a platelet count of 200 × 10³/µL (reference range: 150-400 ×10³/µl).

The patient had no evidence of bleeding or hemolysis. Esophagogastroduodenoscopy and total colonoscopy were performed to rule out gastrointestinal bleeding, with no evidence of active bleeding identified. Esophagogastroduodenoscopy revealed nodular gastritis of the gastric antrum. Histopathological examination of the gastric biopsies was consistent with moderate chronic follicular non-atrophic gastritis with mild activity and confirmed the presence of Helicobacter pylori. No intestinal metaplasia or dysplasia was identified. The rectocolonic mucosa and the terminal ileal mucosa were normal on examination.

The other laboratory investigations revealed hyperferritinemia, with ferritin level of 1665 ng/mL (reference range: 22-275 ng/mL), while folate (vitamin B9) and vitamin B12 levels were within the normal range at 9.3 ng/mL (reference range: 3.2-19.6 ng/mL) and 417 pg/mL (reference range: 180-916 pg/mL), respectively. Hemolysis workup showed no evidence of hemolysis. Total bilirubin was 3 mg/L (reference range: 2-12 mg/L), and indirect bilirubin was 1 mg/L (reference range: <10 mg/L). Haptoglobin was not decreased (3.88 g/L; reference range: 0.14-2.58 g/L), and the direct antiglobulin test (DAT) was negative, arguing against autoimmune hemolytic anemia.

The bone marrow aspirate revealed a richly cellular marrow. Severe erythroblastopenia was observed, with the erythroid lineage accounting for only 1% (reference range: 15%-30%). Megakaryocytes were numerous, and the granulocytic lineage was preserved. A bone marrow biopsy was subsequently performed. The specimen consisted of a 1.7 cm core biopsy with 19 evaluable intertrabecular spaces and showed slightly decreased cellularity for age. Histological examination revealed erythroid hypoplasia, while granulocytic and megakaryocytic lineages were preserved, with no evidence of bone marrow infiltration (Figure 6). Both parvovirus B19 PCR and acetylcholine receptor antibodies were negative. The anemia was attributed to PRCA occurring in the context of a parathymic syndrome. 

**Figure 6:**
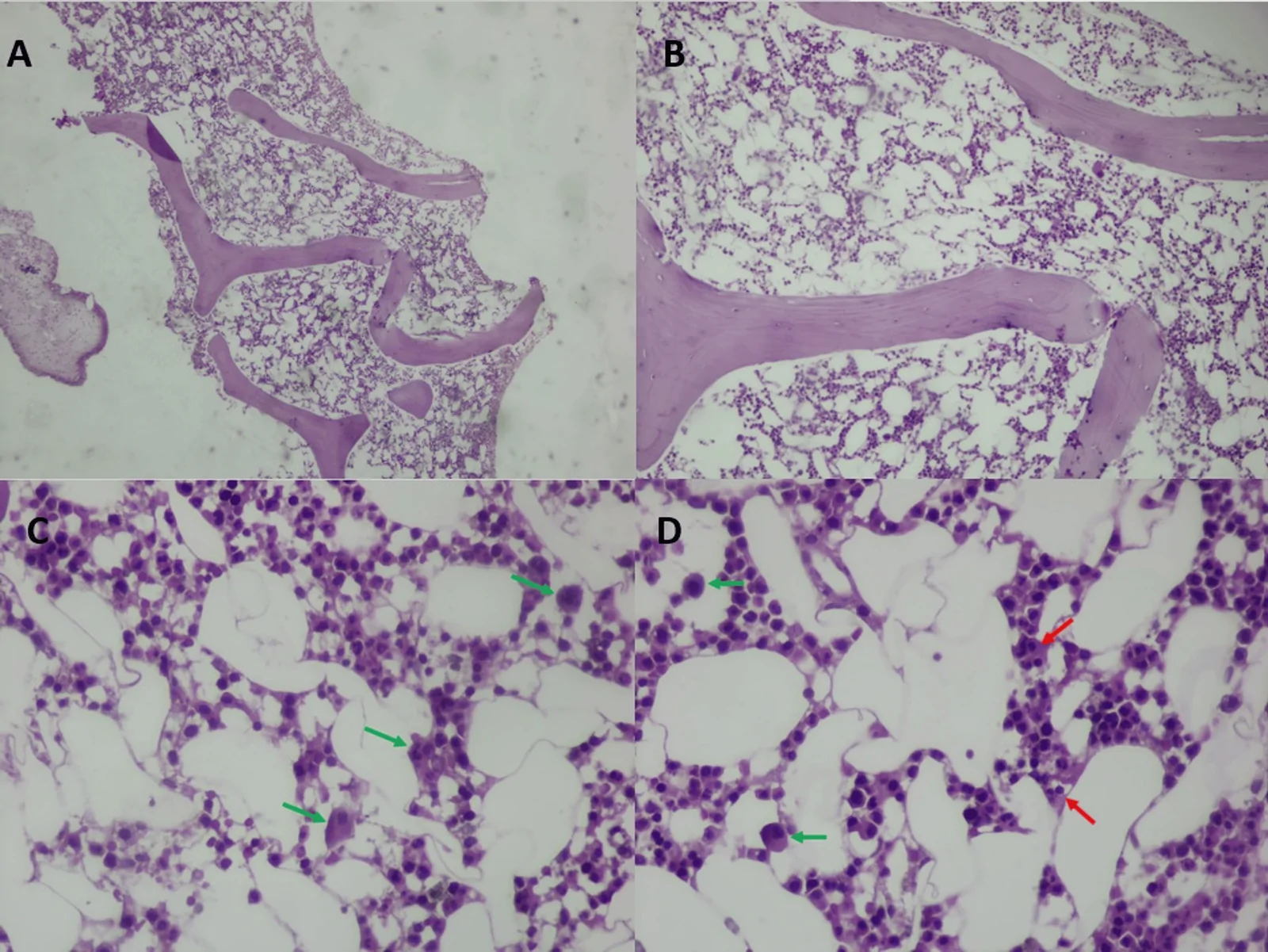
Histological examination of bone marrow biopsy Microscopic views showing preserved granulocytic and megakaryocytic lineages with erythroid hypoplasia (hematoxylin & eosin; A: x40, B: x100, C-D: x400). The green arrows indicate megakaryocytes. The red arrows indicate rare cells of the erythroblastic lineage.

In light of disease progression following second-line chemotherapy and the occurrence of PRCA in this setting, third-line palliative chemotherapy with etoposide (120 mg/m² consecutively on days one to three every 21 days) was recommended during the multidisciplinary team meeting. In parallel, immunosuppressive therapy with prednisone at a dose of 1 mg/kg was initiated to target the underlying autoimmune mechanism of PRCA. Prednisone was selected because corticosteroids are among the first-line treatments for PRCA management [6] and are routinely administered in combination with cytotoxic chemotherapy in many oncologic settings. Although cyclosporine A is also considered a first-line treatment for PRCA [6,7], it was not favored because of the risk of excessive immunosuppression associated with its concomitant use with the planned cytotoxic chemotherapy [8].

Etoposide was administered following the fifth episode of PRCA-related anemia (Figure 5). The patient received a transfusion of four units of PRBCs before the administration of the second course of etoposide because of anemia, with an Hb level of 4.8 g/dL. Before the planned third cycle, the Hb level was 6.4 g/dL. The patient subsequently requested discontinuation of treatment. Etoposide was therefore discontinued, while prednisone was gradually tapered over six weeks before discontinuation. The patient was subsequently monitored with thoracoabdominopelvic CT scans to assess disease progression, while complete blood counts were obtained as needed based on clinical signs of intolerance to anemia to assess the need for transfusion support.

A CT scan performed one month after completion of the two chemotherapy cycles demonstrated stable disease according to the Response Evaluation Criteria in Solid Tumors (RECIST) version 1.1 [9]. Disease stability was maintained on subsequent CT assessments performed four and eight months after the last chemotherapy cycle. At the eight-month follow-up, the thymoma measured 104 mm in its longest diameter, representing the latest available radiological assessment (Figures 7-9).

**Figure 7:**
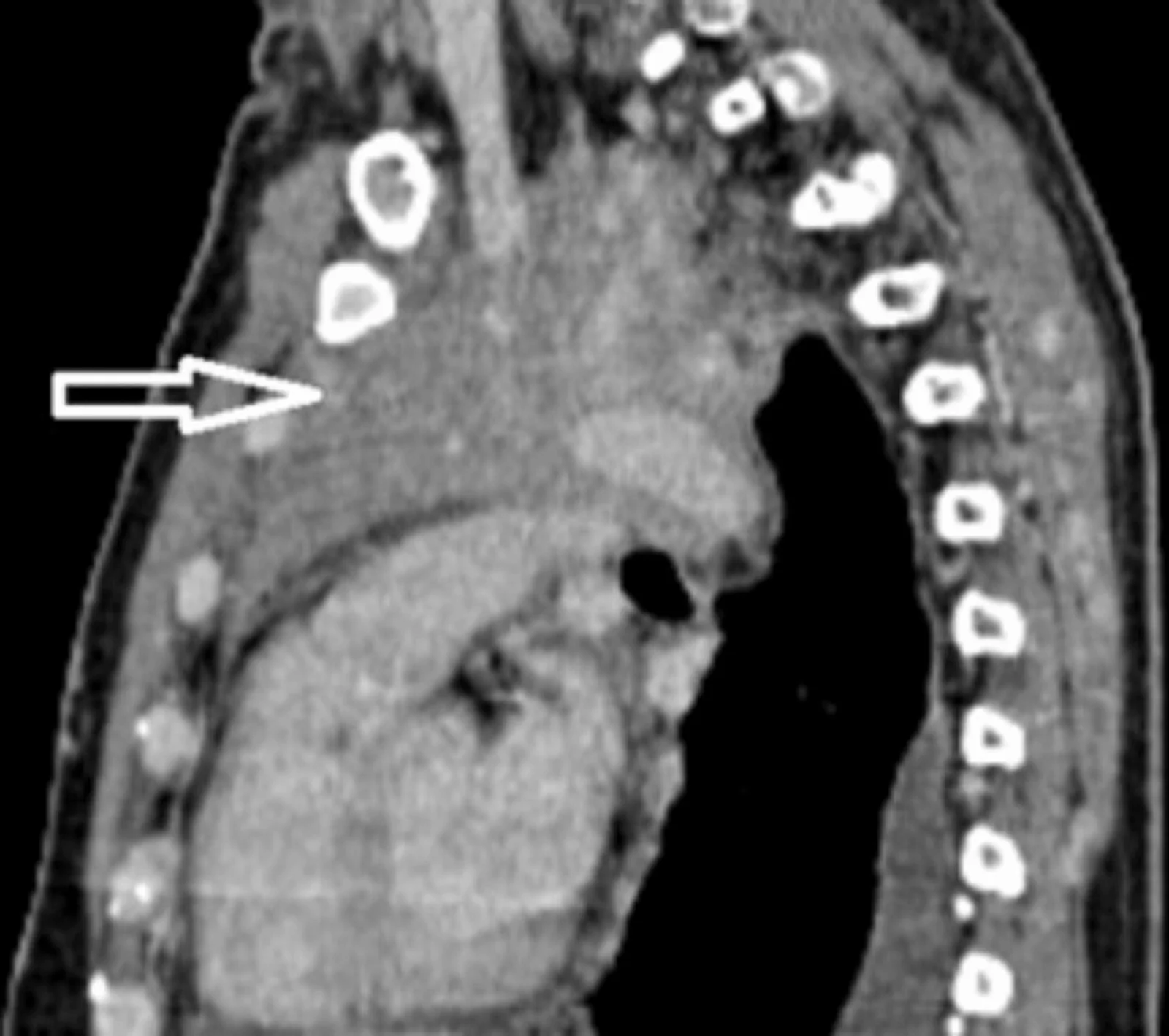
Sagittal CT image of the thymoma at the latest radiological evaluation The arrow points to the thymoma.

**Figure 8:**
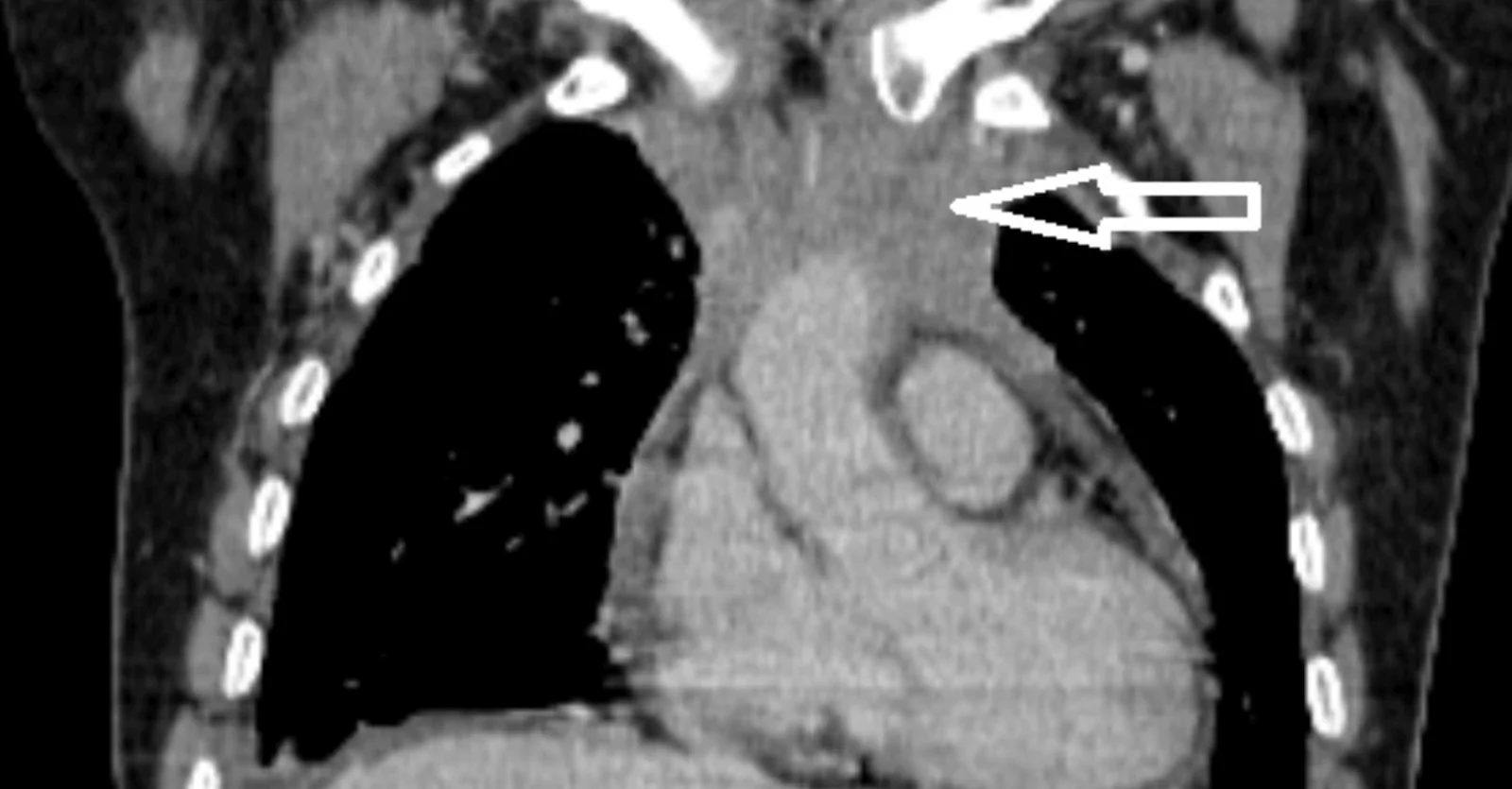
Coronal CT image of the thymoma at the latest radiological evaluation The arrow points to the thymoma.

**Figure 9:**
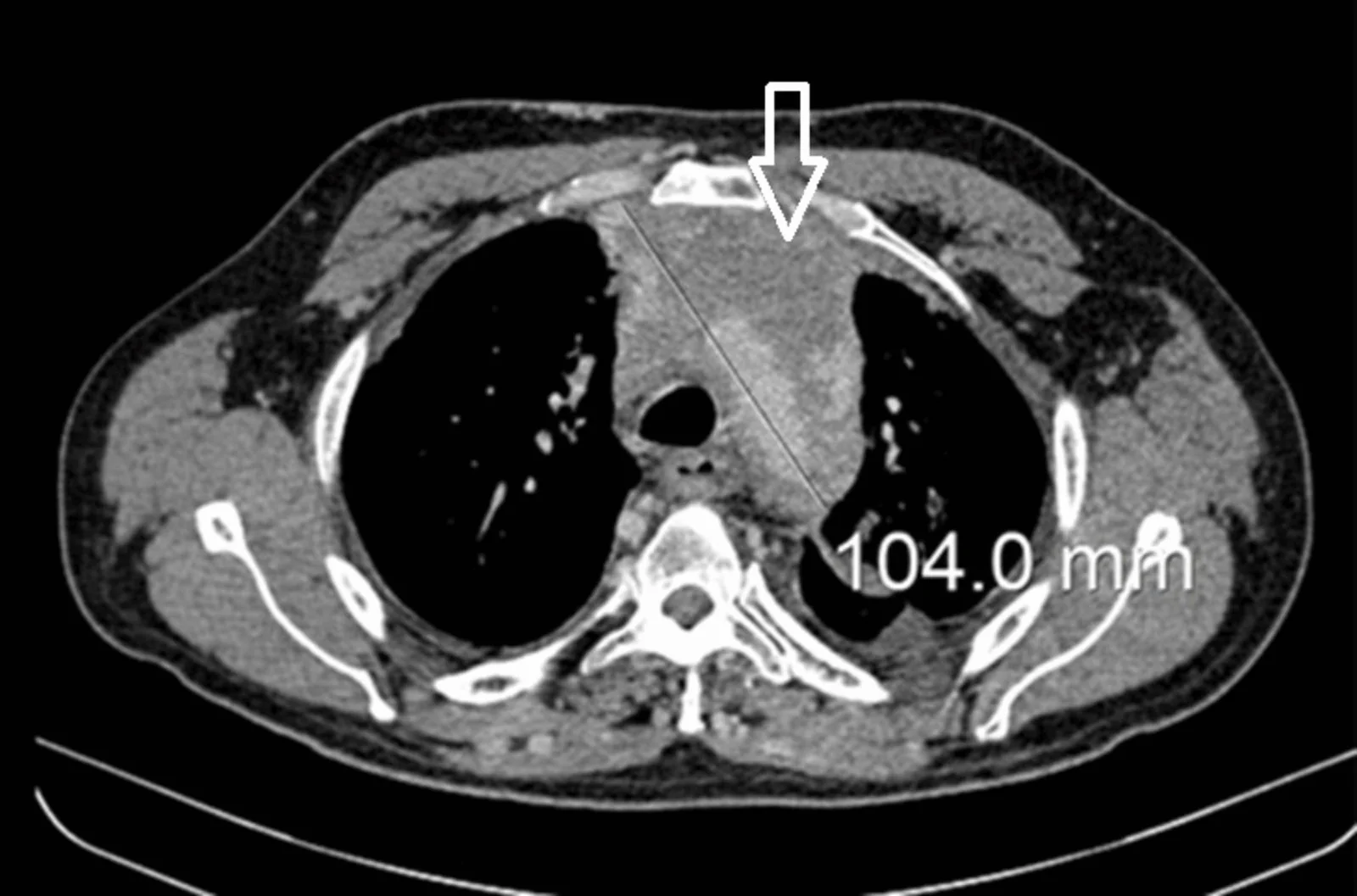
Axial CT image of the thymoma at the latest radiological evaluation The maximum tumor diameter was 104 mm, indicating stable disease according to the RECIST 1.1 criteria. The arrow points to the thymoma. RECIST: Response Evaluation Criteria in Solid Tumors

During the 21.6 months following the second cycle of etoposide, transfusion requirements were monitored, and the patient required 24 transfusion episodes. He received a mean of 2.45 units of PRBCs per transfusion episode (range: one to four units). Transfusions were administered when the Hb level ranged from 4 to 7 g/dL. The median interval between consecutive transfusion episodes was 27 days (IQR, 21-30 days). Thus, no sustained hematologic response to prednisone could be documented, as the patient remained transfusion-dependent throughout follow-up.

## Discussion

Thymomas are malignant tumors characterized by a broad spectrum of clinical behavior, ranging from indolent, slow-growing tumors to aggressive forms with local invasion and distant dissemination. The mainstay of curative treatment for thymoma is complete surgical resection, with postoperative radiotherapy considered in selected patients. In unresectable cases, management typically involves multimodal therapy combining chemotherapy and radiotherapy, delivered either sequentially or concurrently. In locally advanced thymoma, induction chemotherapy may be administered to achieve tumor downstaging before surgical resection [3,10].

Thymomas are epithelial tumors of the thymus frequently associated with autoimmune manifestations, known as parathymic syndromes. Approximately one-third of patients present with autoimmune manifestations at diagnosis. These manifestations result from impaired T-cell differentiation caused by neoplastic thymic epithelial cells and abnormal antigen expression by the tumor. In this context, autoreactive T lymphocytes may be found in the circulation, reflecting disruption of the normal self-tolerant T-cell repertoire, and may lead to B-cell repertoire disruption [2,3].

Myasthenia gravis is the most common autoimmune condition associated with thymoma, occurring in 40% of cases. Other autoimmune disorders may also be present, including hypogammaglobulinemia, PRCA, systemic lupus erythematosus, Sjögren’s syndrome, pemphigus vulgaris, and rheumatoid arthritis. These parathymic syndromes may precede or accompany thymoma or occur following thymectomy. The response of these syndromes to thymectomy is highly variable [1,3].

Pure red cell aplasia is an immune-mediated, non-regenerative anemia characterized by the selective absence of erythroid precursors in the bone marrow, while other hematopoietic lineages remain morphologically normal [2]. Immunosuppressive therapy is frequently required in thymoma-associated PRCA, even after thymectomy. Corticosteroids and cyclosporine A remain the main therapeutic options, with response rates of 77% and 71%, respectively, reported in one study. Other therapeutic options for thymoma-associated PRCA include cyclophosphamide, azathioprine, mycophenolate mofetil, sirolimus, rituximab, and danazol [6].

Findings from a retrospective cohort suggest that surgical resection alone may be insufficient to achieve complete hematologic remission in 12 patients with thymoma-associated PRCA, who all required subsequent adjunctive therapy. After a median follow-up of 26 months, four patients achieved sustained complete remission; they received prednisone alone or in combination with anti-thymocyte globulin or cyclosporine in combination with anti-thymocyte globulin. Two other patients experienced relapse following an initial response to cyclosporine. Treatment-related adverse events included infections, renal failure, and thrombotic thrombocytopenic purpura [11].

Another study reported data on eight patients who underwent surgical resection for thymoma. Two patients had PRCA prior to surgery, in whom no complete remission of anemia was achieved following thymectomy. Six patients received cyclosporine, with or without concomitant corticosteroid therapy, for the treatment of PRCA. After a median follow-up of 54.5 months, three patients achieved complete remission. Reported adverse events associated with cyclosporine included pneumonia and renal failure [7].

A study included seven patients who presented with thymoma and PRCA, either alone or in combination with another hematologic disorder, including Good syndrome or autoimmune hemolytic anemia, and who underwent thymectomy. After a median follow-up of 54 months, four patients achieved remission of PRCA. One patient achieved complete remission following thymectomy alone, while three others achieved complete or partial remission following thymectomy combined with immunosuppressive therapy, including corticosteroids, cyclosporine A, cyclophosphamide, intravenous immunoglobulins, or anti-thymocyte globulin. Among these, one patient achieved complete remission. The authors of this series conducted a systematic review of 60 articles on thymoma-associated PRCA, including a total of 156 patients. In 61% of cases, PRCA achieved remission following thymectomy combined with adjunctive therapies [5].

In the present patient, PRCA complicated the course of a progressive, unresectable thymoma following two lines of chemotherapy, resulting in severe transfusion-dependent anemia and a decline in quality of life. Its occurrence in this context complicates therapeutic management, as it requires both control of tumor progression with further chemotherapy and initiation of immunosuppressive therapy targeting the underlying autoimmune mechanism. This situation increases the risk of infectious complications and limits the feasibility of administering cytotoxic chemotherapy in a transfusion-dependent patient. 

## Conclusions

Thymoma-associated PRCA remains a rare complication that may significantly worsen the clinical course of patients with advanced thymoma. This case underscores the importance of considering immune-mediated hematologic disorders in patients presenting with unexplained severe anemia and highlights the challenges of simultaneously treating progressive malignancy and autoimmune disease. It also emphasizes the value of individualized multidisciplinary management in this complex clinical setting, particularly given the challenges in achieving hematologic recovery in patients with advanced thymoma-associated PRCA.
